# Diode laser‐assisted transcanal endoscopic removal of an aural polyp in the external auditory canal of a dog

**DOI:** 10.1002/vms3.845

**Published:** 2022-05-20

**Authors:** Tomoya Hoshino, Shoko Fukuda, Masahiko Nagata

**Affiliations:** ^1^ Dermatology Service Veterinary Specialists Emergency Centre Saitama Japan; ^2^ Diagnostic Iimaging Service Veterinary Specialists Emergency Centre Saitama Japan

**Keywords:** CT, dog, ear, histopathology, laser, MRI

## Abstract

**Background:**

In humans, aural polyps comprise fibrovascular tissue covered by the respiratory epithelium. Aural polyps with ciliated epithelium are common in cats but are rarely reported in dogs. In a previous case, a mass filled the tympanic cavity alone, and it was surgically removed.

**Objectives:**

To report a case of a canine aural polyp with ciliated epithelium extending from the dorsal tympanic cavity to the external auditory canal with detailed otological features and to demonstrate the usefulness of the transcanal endoscopic procedure (TEP) with a diode laser as a less‐invasive therapy.

**Methods:**

A 12‐year‐old castrated male Cavalier King Charles Spaniel presented with a 6‐month history of unilateral chronic otorrhoea. Video‐otoscope examination revealed a protruding, reddish and soft‐to‐rubbery round mass in the right horizontal ear canal. Computed tomography and magnetic resonance imaging further revealed a smooth mass extending from the dorsal portion of the tympanic cavity into the horizontal part of the external auditory canal. However, it showed no lesions in the dorsal tympanic cavity.

**Results:**

The mass was removed using aural forceps by a traction‐torsion manoeuvre. The suspected base of the mass on the caudal side of the upper tympanic cavity was confirmed by a rigid scope, and it was completely vaporised with a diode laser. Histopathology revealed foci of columnar ciliated epithelium embedded in the connective tissue encapsulated by stratified squamous epithelium. No recurrence was observed at 3 years and 8 months.

**Conclusion:**

We describe a rare case of an aural polyp with ciliated epithelium extending from the upper‐middle ear to the external auditory canal in a dog. The TEP using a diode laser may be a useful minimally invasive treatment option for managing external auditory canal polyps.

## Graphical abstract

This is the first case report describing an aural polyp with cilliated epithelium extending into the auditory canal in a dog with successful outcome using diode laser.

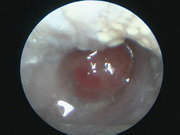

## 1 INTRODUCTION

A polyp is a macroscopic benign projection from mucosal epithelial surfaces, such as the middle ear, colon, cervix, stomach, nose, uterus and throat (Wilcock, 2007). Aural polyps are histologically confirmed by a core of loosely arranged fibrovascular tissue covered by columnar ciliated epithelium (Wilcock, 2007). They are associated with chronic inflammation in the middle ear, and cholesteatomas are considered one of the aetiological causes in humans (Gliklich et al., 1993; Milroy et al., 1989). The terms ‘aural polyp’ or ‘inflammatory polyp’ in the ear can be widely used in veterinary medicine for any fibrovascular tissue projection with or without respiratory epithelium (Wilcock, 2007); polyps originating from the middle ear are commonly seen in cats. However, they are uncommon in dogs (Blutke et al., 2010; Greci & Mortellaro, 2016; Pratschke et al., 2003). There are only two detailed descriptive reports on aural polyp in dogs (Blutke et al., 2010; Pratschke et al., 2003). Blutke et al. (2010) reported the case of a dog with a typical aural polyp, as seen in humans and cats. A polyp filled the middle ear without extending into the external auditory canal. It was histologically confirmed by the presence of the overlying surface layer of the respiratory epithelium. In this case, surgical resection of an aural polyp using ventral bulla osteotomy (VBO) was performed. This approach led to higher morbidity than simple traction; however, it carried a lower risk of recurrence (Greci & Mortellaro 2016). Aural polyps in cats are removed by per‐endoscopic trans‐tympanic traction as a non‐surgical approach (Greci et al., 2014); this is minimally invasive. However, in this instance of simple repeated grasping and pulling to debulk the mass, a portion of the stalk remnant may remain, which increases the risk of polyp recurrence (Greci & Mortellaro, 2016). Herein, we report the case of a dog with an aural polyp comprising of ciliated epithelium that extended from the middle ear to the external auditory canal, where the transcanal endoscopic procedure (TEP) using a diode laser resulted in a successful outcome.

## 2 CASE REPORT

### 2.1 Case history

**FIGURE 1:**
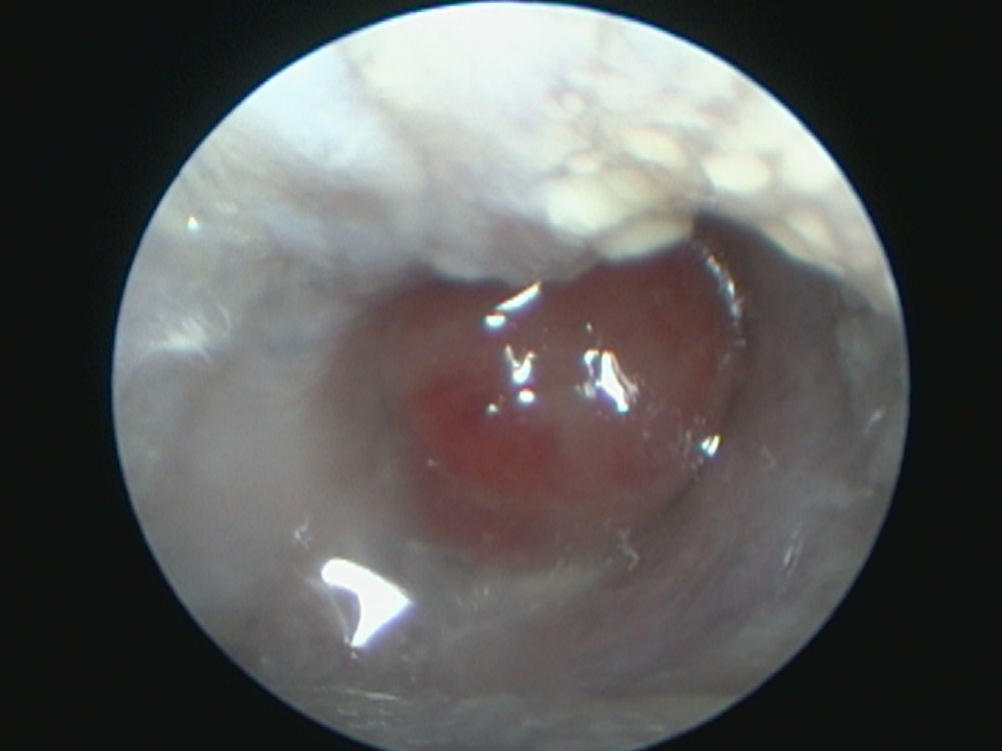
Video‐otoscope finding. A reddish‐round nodule can be seen in the horizontal external auditory canal

A 12‐year‐old castrated male Cavalier King Charles Spaniel (CKCS) was referred to Dermatology at the Veterinary Specialists Emergency Centre with a 6‐month history of unilateral chronic otorrhoea in the right ear. This case had been treated with antimicrobials, including minocycline and metronidazole; however, there was no improvement. The dog was clinically healthy and had no neurological signs. A video‐otoscope (Karl Storz GmbH & Co. KG, Tuttlingen, Germany, or Asuka Medical Inc., Kyoto, Japan) examination revealed a protruding, reddish and soft‐to‐rubbery round mass in the right external auditory canal with yellowish purulent ear discharge (Figure 1). The differential diagnoses included granulomas, cholesteatomas and tumours, such as ceruminous gland adenomas.

### 2.2 Diagnostic procedure

**FIGURE 2:**
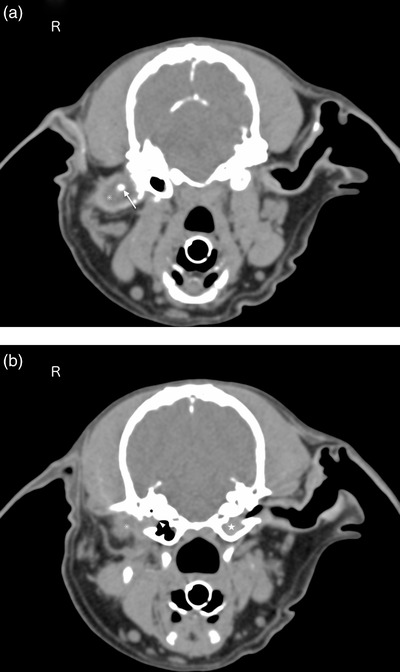
Computed tomography of the skull. (a) A smooth mass is seen in the horizontal external auditory canal in the right ear (asterisk). The distal portion of the mass is homogeneously enhanced. In contrast, the proximal portion is heterogeneously enhanced with partial mineralisation (white arrow). (b) A mass is seen extending from the dorsal portion of the tympanic cavity (white arrowhead) but not into the ventral portion of the tympanic cavity. Coincidentally, the middle ear effusion is seen in the contralateral left tympanic cavity (white star)

**FIGURE 3:**
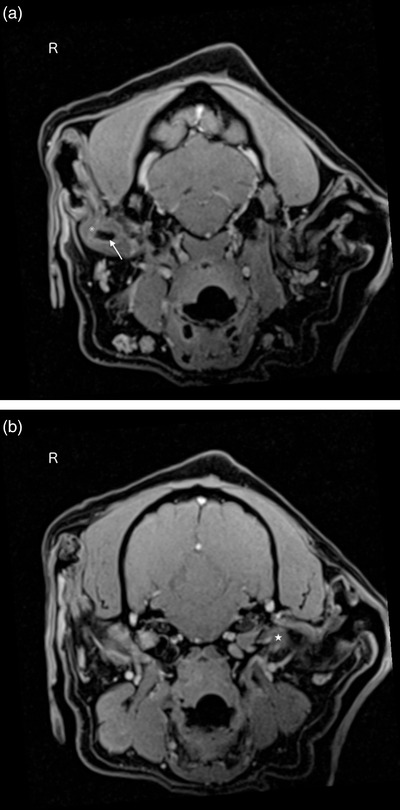
Magnetic resonance imaging of the skull. (a) The mass is isointense on T2‐weighted imaging (T2WI) and fluid‐attenuated inversion recovery (FLAIR) and slightly hyperintense in the right external auditory canal on T1‐weighted imaging (T1WI) compared to the grey matter (asterisk) and has a hypointense core on all sequences with susceptibility of an artefact on T2* (observed T2WI), which is consistent with mineralisation (white arrow). (b) In the contralateral tympanic cavity, there is a T2WI/ FLAIR and T1WI hyperintense material, consistent with a high protein fluid (white star)

Microbial culture and sensitivity testing of the discharge from the right ear were performed to evaluate the presence of antimicrobial‐resistant bacteria due to the long‐term use of several antibiotics. Methicillin‐sensitive *Staphylococcus pseudintermedius* was isolated later; however, its pathogenicity could not be determined since ear cytology was not performed in this case. Complete blood cell counts and a serum chemistry panel were performed prior to diagnostic imaging under general anaesthesia; these tests, however, did not show any abnormalities. Computed tomography (CT) (Somatom Emotion 16, Siemens Healthcare, Tokyo, Japan) revealed a smooth mass extending from the dorsal portion of the tympanic cavity into the horizontal external auditory canal, with a homogeneously enhanced distal portion and heterogeneously enhanced and partially mineralised proximal portion. The horizontal external auditory canal had been focally filled with the mass and fluid (Figure 2), and no mass lesions were observed in the ventral portion of the tympanic cavity. Magnetic resonance imaging (MRI) (MAGNETOM Essenza 1.5T, Siemens Healthcare, Tokyo, Japan) revealed the mass that was isointense on T2‐weighted imaging (T2WI) and on fluid‐attenuated inversion recovery (FLAIR). Moreover, it was slightly hyperintense on T1‐weighted imaging (T1WI) compared to the grey matter, and it had a hypointense core on all sequences with a suspected artefact on T2* (observed T2WI), consistent with mineralisation. In the contralateral left tympanic cavity, hyperintense material was coincidentally noted on T2WI/FLAIR and T1WI, consistent with high protein fluid suggestive of secretory otitis media (Figure 3).

### 2.3 Treatment

**FIGURE 4:**
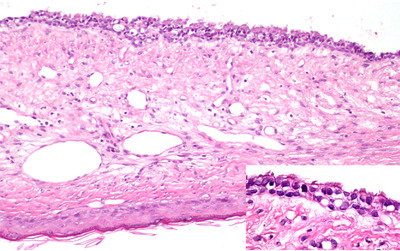
Representative photomicrograph of the middle ear polyp. The proliferation of connective tissues is seen with ciliated epithelium (insert). Haematoxylin and eosin stain

The mass was removed using 9 cm‐long aural forceps (Micro‐curette, SHIN‐MEDICO Inc., Chiba, Japan) for traction‐torsion via the TEP under general anaesthesia. The external auditory capacity showed no apparent pathologic changes after removing the polyp. The suspected base of the mass around the epitympanic recess on the caudal site of the upper tympanic cavity was confirmed by a rigid scope (AES‐30A, Asuka Medical Inc.) and completely vaporised using a diode laser (DLV‐20, Asuka Medical Inc.). In this operation, the ear was flushed using a soft feeding tube with neutral electrolysed water. Histopathological evaluation revealed a polypoidal mass with a thick fibrovascular stalk (Figure 4). The lesion's surface was encapsulated by stratified squamous epithelium, and a widely dilated ductal structure with columnar ciliated epithelium embedded in the connective tissue was observed accordingly. Partial central calcification at the base area of the polyp was also seen with mild to moderate infiltration of the stroma by lymphocytes, plasma cells and a few neutrophils. Based on these findings, an aural polyp was confirmed in this case. Postoperatively, 0.5 mg/kg oral prednisolone (Predonine tablets, Shionogi & Co., Ltd., Osaka, Japan) once daily was prescribed to prevent inflammatory reaction to intensive ear flushing and laser‐assisted transcanal endoscopic ablation. Cephalexin (Rilexpet’ A, Virbac Japan Co., Ltd., Osaka, Japan) at 20 mg/kg twice daily was also administered orally to manage secondary infections. In‐clinic middle ear flushing was performed using a 4‐Fr feeding tube (Atom Indwelling Feeding Tube for Infant, Atom Medical Corporation Inc., Saitama, Japan) with neutral electrolysed water twice at a week's interval without general anaesthesia or sedation to remove the debris due to otitis media and laser ablation. This dog had an intact tympanic membrane without neurologic signs on the left side; hence we decided to follow the clinical course without additional treatments for possible secretory otitis media. Follow‐up at 3 years and 8 months after treatment confirmed no recurrence of polyp, bacterial otitis or primary secretory otitis media.

## 3 DISCUSSION

We report a case of a dog with an aural polyp extending from the upper‐middle ear to the external auditory canal treated with traction and transendoscopic laser ablation. Blutke et al. (2010) reported an aural polyp arising from the Eustachian tube in a 10‐year‐old male dog. MRI revealed a hyperintense soft tissue mass that filled the middle ear cavity; the polyp was histologically composed of a fibrous connective tissue stroma covered by respiratory epithelium. This seemed to be a classic form of an aural polyp, similar to those reported in feline and human cases. Another article reported a mass in the external auditory canal originating from the tympanic bulla in four dogs and multiple smaller polypoid growths originating from the tympanic membrane in one dog (Pratschke et al., 2003). However, there was no columnar ciliated epithelium, and the fibrovascular stroma was covered by a stratified squamous epithelial layer. Additionally, marked dilation and hyperplasia of ceruminous glands were identified within and around the polyp in two cases. Furthermore, all dogs had radiographic evidence of enlargement of the bullae in the affected bulla cavity. Imai et al. (2019) reported a protruding white to pale pink mass in the external auditory canal in 7 of 11 dogs with cholesteatoma confirmed by histopathological evaluation. In their cases, the histological findings of the mass were compatible with those of polyps reported by Pratschke et al. (2003). In one retrospective study on aural polyps in humans, two categories of polyps were described accordingly: polyps associated with cholesteatoma and those unrelated to cholesteatoma (Milroy et al., 1989). It is assumed that aural inflammatory polyps and polypoid masses are not single entities; as in humans, cholesteatoma‐associated polypoid growth is a differential diagnosis of aural polyps in dogs. In the present case, the histopathological evaluation revealed a mass with columnar ciliated epithelium embedded in the connective tissue encapsulated by stratified squamous epithelium. Furthermore, neither CT nor MRI revealed any findings suggestive of cholesteatoma, including bone changes at the contour of the tympanic bulla, osteolysis, osteoproliferation and osteosclerosis, expansion of the tympanic cavity or sclerosis or osteoproliferation of the ipsilateral temporomandibular joint and the paracondylar process (Imai et al., 2019; Travetti et al., 2010). We believe that this is the first description of an aural polyp with ciliated epithelium extending from the upper‐middle ear to the external auditory canal in a dog that resembled feline and human cases.

The polyp in the present case had partial central calcification. Similar clinical and histological findings have been reported in human aural polyps (Sogebi, 2012). A few foci of calcification in the polyp were also observed in a previous case in a dog (Blutke et al., 2010). The present case had a 6‐month history of otorrhoea. It is suspected that a long‐standing inflammatory process in the middle ear led to this pathologic change. The difference in epithelial lining may have reflected differences in aetiology, degree and chronicity of concomitant inflammation, as well as different anatomical sites of origin of aural polyps (Blutke et al., 2010). An aural polyp is considered a non‐specific pathological sequela or complication of chronic otitis media in humans; conversely, polyps can also cause otitis media (Gliklich et al., 1993). CKCS is a breed known to be predisposed to primary secretory otitis media; the concurrent otitis media observed in the contralateral tympanic cavity suggests subclinical chronic inflammation in the middle ear.

In the case reported by Blutke et al. (2010), the aural polyp was removed surgically via ventral bulla osteotomy, and other reported canine polyps to date have also been surgically resected similarly. In contrast, most feline polyps reported have been removed via non‐surgical means. Regardless of the approach, postoperative complications can occur (Greci & Mortellaro, 2016). Polyps are clinically benign and rarely life‐threatening lesions; therefore, a less‐invasive approach should be considered where feasible (Imai et al., 2019). Traction‐avulsion is the simplest means; however, a portion of the stalk may remain, increasing the risk of polyp recurrence (Greci & Mortellaro, 2016). Per‐endoscopic trans‐tympanic traction using small forceps or curettes can remove the residual portion and reduce the recurrence rate; however, this procedure is time‐consuming (Greci et al., 2014). Carbon dioxide laser ablation of polyps is another promising technique for aural polyp removal (Greci & Mortellaro, 2016). Imai et al. (2019) described diode laser ablation of the middle ear tissue for non‐surgical management of cholesteatoma. A diode laser under a video‐otoscope observation has also been used to excise a complex ceruminous adenoma in the external auditory canal (Usui et al., 2015). In the present case, the base of the polyp was vaporised by the diode laser, and a successful outcome was subsequently obtained. Recently, diode lasers have been introduced in human ear surgeries. Laser myringotomy reduces intraoperative bleeding, postoperative infection rate, the risk of inducing cholesteatoma, and collateral damage to the surrounding healthy tissues, thereby avoiding complications (Zong et al., 2019). However, the laser operation has to be carefully handled under rigid endoscopic view since there are potential risks. Structures in the middle ear area, including the auditory ossicles, are complicated, and the anatomical details are not clearly visible under pathologic change. Further studies are needed to estimate the effectiveness of this treatment in middle ear lesions.

## 4 CONCLUSIONS

A dog had an aural polyp with ciliated epithelium extending from the upper‐middle ear to the external auditory canal, which was confirmed by both diagnostic imaging and histopathology. It was successfully removed using transendoscopic traction plus laser ablation of the base without a traditional surgical approach. No recurrence was observed at 3 years and 8 months. TEP using a diode laser seems to be valuable as a less‐invasive approach.

## CONFLICT OF INTEREST

No conflicts of interest have been declared.

## AUTHOR CONTRIBUTIONS

Tomoya Hoshino: conceptualisation; data curation; resources; validation; visualisation; writing – original draft. Shoko Fukuda: validation; visualisation; writing – original draft. Masahiko Nagata: conceptualisation; data curation; resources; supervision; validation; writing – review & editing.

## ETHICS STATEMENT

The authors confirm that the ethical policies of the journal, as noted on the journal's author guidelines page, have been adhered to. No ethical approval was required as this is a retrospective case report with no original research data.

## STUDY PRESENTATION

This study has been presented in Asia Meeting of Animal Medicine Specialties in 2019.

### PEER REVIEW

The peer review history for this article is available at https://publons.com/publon/10.1002/vms3.845.

## Data Availability

Data openly available in a public repository that issues datasets with DOIs
